# Enlightening the invisible: Applications, limits and perspectives of intraoperative fluorescence in neurosurgery

**DOI:** 10.1016/j.bas.2024.103928

**Published:** 2024-10-10

**Authors:** Giulia Cossu, Tuan Le Van, Luc Kerherve, Sayda A. Houidi, Edouard Morlaix, Florent Bonneville, Renan Chapon, Olivier Baland, Catherine Cao, Maxime Lleu, Walid Farah, Ahmed El Cadhi, Jacques Beaurain, Thiebaud Picart, Bin Xu, Moncef Berhouma

**Affiliations:** a Department of Neurosurgery, University Hospital of Lausanne and University of Lausanne, Lausanne, Switzerland; b Department of Neurosurgery, University Hospital of Dijon Bourgogne, Dijon, France; c Department of Neurosurgery, Groupe Hospitalier Est, Hôpital Neurologique Pierre Wertheimer, Hospices Civils de Lyon, Bron, France; d Université Claude Bernard Lyon 1, 43 Bd du 11 Novembre 1918, Villeurbanne, France; e Cancer Research Centre of Lyon (CRCL), INSERM 1052, CNRS 5286, 28 Rue Laennec, Lyon, France; f Department of Neurosurgery, Huashan Hospital, Fudan University, Shanghai, China; g Functional and Molecular Imaging Team (CNRS 6302), Molecular Chemistry Institute (ICMUB), University of Burgundy, France

**Keywords:** 5-ALA, Fluorescence, Fluorophores, Fluorescein sodium, Indocyanine green, Microneurosurgery

## Abstract

**Introduction:**

The introduction of intraoperative fluorophores represented a significant advancement in neurosurgical practice. Nowadays they found different applications: in oncology to improve the visualization of tumoral tissue and optimize resection rates and in vascular neurosurgery to assess the exclusion of vascular malformations or the permeability of bypasses, with real-time intraoperative evaluations.

**Research question:**

A comprehensive knowledge of how fluorophores work is crucial to maximize their benefits and to incorporate them into daily neurosurgical practice. We would like to revise here their applications and clinical relevance.

**Material and methods:**

A focused literature review of relevant articles dealing with the versatile applications of fluorophores in neurosurgery was performed.

**Results:**

The fundamental mechanisms of action of intraoperative fluorophores are enlightened, examining their interactions with target tissues and the principles driving fluorescence-guided surgery. The clinical applications of the principal fluorophores, namely fluorescein sodium, 5-ALA and indocyanine green, are detailed, in regards to the management of vascular malformations, brain tumors and pathologies treated through endoscopic endonasal approaches.

**Discussion and conclusion:**

Future perspective dealing with the development of new technologies or of new molecules are discussed. By critically assessing the efficacy and applications of the different fluorophores, as well as charting their potential future uses, this paper seeks to guide clinicians in their practice and provide insights for driving innovation and progress in fluorescence-based surgery and research.

## Highlights

• Fluorophores maximize tumor removal and improve outcomes of vascular malformations.
• FNa and ICG videoangiography have complementary advantages.
• 5-ALA is the gold standard for fluorescence-guided surgery for high-grade gliomas.
• FNa may be a valid alternative to 5-ALA for glioma surgery and for other tumors.
• FNa and ICG may be useful tools in performing endoscopic endonasal procedures.

## 1 Introduction

Neurosurgical procedures require precise knowledge of anatomical structures and delineation of pathological entities to optimize patient outcomes. Since the early 2000s, intraoperative fluorophores have emerged as promising intraoperative tools, improving real-time visualization of pathological tissue and enhancing surgical accuracy (Picart et al., 2019; Schupper et al., 2021). The evidence for the use of fluorescence-guided surgery (FGS) has thus gradually increased. A comprehensive understanding of the mechanisms of action of intraoperative fluorophores and of their clinical utility is the premise to harness their full potential and integrate them effectively into neurosurgical workflows. The fundamental principles underlying the use of fluorophores are based on light absorption by the fluorophore itself, which thus switches to an excited energy state. Subsequently, returning to its resting state, the excess energy is released as light (*fluorescence*), with longer wavelengths than the lights absorbed, thus allowing fluorescence detection (*wavelength shift*) (Schupper et al., 2021). We can differentiate two main categories of fluorophores according to the wavelengths of emitted light: those visible under white light and those requiring a filter to be visualized.

Scular neurosurgery and neuro-oncology are the two main fields of application of intraoperative fluorophores. Vascular neurosurgery necessitates a careful balance between the necessity to properly treat vascular malformations and a meticulous assessment of integrity of normal vessels, to mitigate the risk of ischemic complications. Thus, intraoperative fluorophores offer a means to: (1) identify the abnormal vasculature and assess the complete exclusion of vascular malformations and (2) to verify the vascular permeability during surgery in real time, providing invaluable insights into vessel patency. In oncological neurosurgery, the precise delineation of tumor margins is fundamental to achieve maximal resection while preserving critical neural structures. Nevertheless, identification of tumor boundaries may pose a significant challenge with tumors lacking distinct demarcations and morphologically similar to healthy tissue. Consequently, relying solely on visual inspection for assessing the extent of resection can be unreliable (Albert et al., 1994; Hadjipanayis et al., 2015) and the use of intraoperative fluorophores use may facilitate tumor visualization and thereby enhance the extent of resection (Ahrens et al., 2022).

Furthermore, the role of fluorescence in endoscopic endonasal procedures has been largely investigated during the last decade (Chang et al., 2019; Lakomkin et al., 2021). Fluorophores can have different applications that are proper to this kind of procedure and, beside their classical application in maximizing surgical resections, they can be used to identify the site of CSF leak, to preserve the pituitary stalk and the pituitary gland (Litvack et al., 2012; Vergeer et al., 2022) and to visualize vascular structures, thus limiting the risk of postoperative endocrinological and vascular morbidity (Cossu et al., 2020).

This review will explore the principles governing FGS and the interaction of intraoperative fluorophores with target tissues, to critically evaluate their usefulness and application in different domains of neurosurgery. Challenges such as administration modalities, tissue penetration depth, specificity, and potential off-target effects necessitate careful consideration and we will discuss these limitations and explore potential strategies to address them, while highlighting emerging technologies and future directions in the field.

## 2 Material and methods

A comprehensive literature review of published articles dealing with clinical applications of different fluorophores in cranial neurosurgery was performed on PubMed and Embase databases. The search terms were chosen and combined with Boolean operators to capture the breadth of the topic including the following keywords: *“fluorophores”, “fluorescence-guided surgery”, “cranial neurosurgery”, “brain tumors”, “vascular neurosurgery”, “vascular malformations”.* More specific information on each fluorophore was retrieved using the terms: *“fluorescein sodium”, “5-aminolevulinic acid”, “indocyanine green”* combined with the previously detailed keywords. The reference lists of relevant articles were also manually reviewed to identify additional sources. No language restrictions were applied and articles published after 1990 were preferred.

We included reviews, clinical articles and case reports published in peer-reviewed journals and this narrative review was thus structured in different sections according to the type of fluorophore considered, while detailing the specific clinical applications, mechanisms of action, way of use with posology and timing of administration, along with benefits, limitations and safety profile. According to literature data, comparative analyses of the efficacy of the fluorophore in improving surgical outcomes were reported.

## 3 Results

The current knowledge on the subject is summarized into subchapters corresponding to the specific fluorophore analyzed. The main characteristics of each fluorophore are summarized in Table 1, while their posology and timing of administration, along with the advantages and disadvantages of each product are reported in Table 2.

• **Fluorescein Sodium (FNa)**

**Table 1:** Summary of the different mechanism of action, pharmacodynamic and pharmacokinetic for the three most common fluorophores used in neurosurgery.

	Mechanism	Filter	Excitation wavelengths	Emission wavelengths	Metabolism	Half-life	Side effects
**FNa**	1. Binds to plasma proteins 2. Accumulates in areas of BBB disruption	*NO* but YELLOW 560 filter can be used	460–500 nm	540 and 690 nm (yellow/green light)	Liver	20 min (conjugated form about 4h)	Skin, mucosa and urine staining (24–48h) Rare: cardio-pulmonary reactions/seizure
**5-ALA**	Metabolites accumulate in tumoral cells	*YES:* BLUE 400 filter	375–440 nm (blue light)	640–710 nm (red light/UV)	Tumor cells, liver, kidneys or skin	1–3 h	sunlight-induced erythema Blood count alterations/Hepatic enzymes elevation CI: porphyria, pregnancy, kidney and hepatic diseases
**ICG**	Binds to plasma proteins	*YES:* IR-800 filter/NIR camera	805 nm (NIR)	820–860 nm (NIR)	Liver	3–5 min	Rare: nausea/skin rash

Abbreviations.

5-ALA: 5-Aminolevulinic acid.

CI: contraindications.

FNa: Fluorescein Sodium.

ICG: indocyanine green

NIR: near-infrared.

nm: nanometers.

**Table 2:** Summary of the different applications of fluorophores in neurosurgery, along with the posology, timing of administration, list of advantages and disadvantages.

Application	Agent	Aim	Way of administration	Dosage	Timing of administration	PRO	CONS
**Tumoral surgery**	FNa	Tumor visualization	IV	2–5 mg/kg with filter 10–20 mg/kg with no filter	30 min before skin incision	Safe Applied to resection of different CNS tumors Pediatric application possible	Nonspecific (false positives)
	5-ALA	Tumor visualization	Oral	20 mg/kg	3–4 h before surgery	Gold standard for selective staining of high-grade gliomas Detection of anaplastic foci in gliomas	Expensive Not useful with other CNS tumors Coordination with the OR required (timing of administration) Frequent switch between fluorescent image and white light is necessary Side effects possible
	ICG	Vessels visualization	IV: bolus	0.2–0.5-mg/kg	1 min before (arterial phase)	Visualization of normal and tumoral vessels	
		Tumor visualization	IV: second window technique	5.0 mg/kg	24 h pre-op	Safe Applied to resection of different CNS tumors	Coordination with the OR required (timing of administration)
**Vascular surgery**	FNa	Vessels visualization	IA	10 ml of 0.01–0.02% in bolus; 0.001–0.002 mg	1–2 min before (arterial phase)	Quick washout (<1min) Better visualization of small perforating vessels than ICG	Invasive (arterial access required: ICA)
			IV	2000 mg or 1–1.5 mg/kg body weight	4–8 min before (arterial phase)	No supplementary arterial access required Better visualization of small perforating vessels than ICG	Slower washout (10 min) Higher doses required and can stain the vessels
	ICG	Vessels visualization	IV	0.2–0.5-mg/kg	1 min before (arterial phase)	Quick washout with multiple administrations possible Flow measurements	No contemporary anatomy visualization Chromatic aberrations in deep operative fields
**Endoscopic endonasal surgery**	FNa	Identification of CSF site leak	IT	25 mg	30 min before	Helpful in difficult cases to identify the leak	Lumbar tap/drain is necessary
		Tumor visualization	IV	5–10 mg/kg	10 before durotomy	Safe It may differentiate PitNET from scar tissue	
		Normal pituitary visualization			Soon after durotomy	Helpful in preserving normal tissue	
	5-ALA	Tumor visualization	Oral	20 mg/kg	3–4 h before surgery		Not efficient Expensive
	ICG	Vessels and normal tissue visualization	IV	12.5–25 mg	1min before the visualization	Safe Multiple injections possible Useful to evaluate: - the viability of the nasoseptal flap - the position of the ICA - to differentiate normal pituitary gland/stalk from tumors No impact from the hormonal expression of the PitNET Endoscopic fluorescence is prolonged	Clear operative field required Dedicated endoscope required Lack of simultaneous visualization of fluorescent vs non-fluorescent image
	OTL-38	Tumor visualization	IV	0.025 mg/kg in infusion	2–4 h before surgery	Specific for non-functional PitNET over-expressing FRα	Not clear its role for other pathologies Coordination with the OR required (timing of administration)

Abbreviations.

5-ALA: 5-Aminolevulinic acid.

CNS: central nervous system.

FNa: Fluorescein Sodium.

FRα: folate receptor α

ICG: indocyanine green

IA: intraarterial.

ICA: internal carotid artery.

IT: intratechal.

IV: intravenous.

min: minutes.

Kg: kilograms.

OR: operative room.

PitNET: pituitary neuroendocrine tumors.

Fluorescein sodium (FNa) was first applied in ophthalmology, where it gained prominence for assessing retinal blood flow in diabetic retinopathy in the mid-20th century (Russell et al., 1966; Norton and Gutman, 1965), and it found further applications in visualizing neoplastic tissue in the 1940s (Moore, 1947; Moore et al., 1948). FNa emits a yellow-green fluorescence visible under white light, with an excitation falling within the range of 460–500 nm (nm) and emission between 540 and 690 nm (Hohne et al., 2017). Upon intravenous administration, FNa loosely binds to plasma proteins (80%) and metabolism primarily occurs via glucuronidation in the liver, with rapid clearance by the kidneys, leading to almost complete excretion within 24 h (Zhao et al., 2019). Its mechanism of action involves a “mechanical” and non-cell-specific accumulation in extracellular areas of the central nervous system (CNS) where the blood-brain barrier (BBB) is disrupted (Bomers et al., 2020; Schipmann et al., 2019), potentially extending its utility to different applications (Ahrens et al., 2022; Falco et al., 2019) but impairing its specificity. When compared to other fluorophores, FNa offers several advantages, including lower costs, ease of use and absence of relevant side effects. In clinical practice, FNa can be administered intravenously at the time of anesthesia and exhibits maximal fluorescence within three to 4 h. The YELLOW 560 nm filter of the operating microscope may enhance its visualization and reduce the posology administered (<5 mg/kg), although with higher doses (around 20 mg/kg), darkening of the operating room is not necessary and fluorescence can be visualized without specific filters. Adverse effects are rare and may be light, including temporary skin, mucosa and urine staining in the first 24–48h after surgery but the phenomenon seems self-limited and without sequelae. In rare cases (1:1900), more severe complications can be observed, such as cardiac effects, respiratory reactions, or seizures and they seem to be related to the use of higher doses (Tanahashi et al., 2006; Dilek et al., 2011) and intrathecal injections. Death was reported in 1 case out of 222,000 (Yannuzzi et al., 1986).

In vascular neurosurgery, FNa finds different application in performing videoangiography, assisting in the treatment of vascular malformations, while it may help in identifying tumoral tissue in oncological procedures and in evaluating the site of cerebrospinal fluid (CSF) leaks during endoscopic endonasal procedures, as further detailed.

### 3.1 Vascular neurosurgery

The application of FNa in vascular neurosurgery has received increased attention in recent years (Zhao et al., 2019), but its initial use dates back to a pioneer study conducted in 1971, where FNa was utilized during surgical excision of an arteriovenous malformation (AVMs) (Feindel et al., 1971). However, its utilization remained largely unexplored until its relevance was highlighted in 2007 (Suzuki et al., 2007). Since then, there has been a gradual but steady increase in studies employing FNa videoangiography (Kakucs et al., 2017; Rey-Dios and Cohen-Gadol, 2013; Ichikawa et al., 2014; Matano et al., 2017; Lane et al., 2015; Hashimoto et al., 2017; Ito et al., 2019; Serrano-Rubio et al., 2018), as it proved to be useful in monitoring blood flow during aneurysm clipping, in assessing the permeability of perforating arteries, and in evaluating AVM morphology and microvascular anastomosis patency (Zhao et al., 2019) (Little et al., 2019). Both intraarterial and intravenous (iv) administration routes were described, each offering specific benefits (Zhao et al., 2019). Intraarterial injection results in faster contrast appearance and higher-intensity fluorescence, requiring a lower dosage (10 ml of 0.01–0.02% in bolus; 0.001–0.002 mg) (Kuroda et al., 2013) compared to intravenous injection (2000 mg or 1–1.5 mg/kg body weight) (Zhao et al., 2019; Rey-Dios and Cohen-Gadol, 2013; Kuroda et al., 2013; Misra et al., 2017). However, a supplementary arterial access should be obtained with the risk of related complications, as the best administration is performed directly in the carotid artery. On the other side, the higher doses used for the intravenous route can provoke an unspecific vessel wall staining.

Various optical tools have been successfully used for FNa videoangiography, including conventional operative microscopes with specialized filter sets, light-emitting diode probes, laser-illumination microscopes, and endoscopes (Zhao et al., 2019). The advantages of FNa videoangiography are the three-dimensional visualization of surrounding anatomy with the possibility of real-time surgical manipulation (Zhao et al., 2019; Kucukyuruk et al., 2021), and the improved visualization of small vascular structures within deep operative fields (Suzuki et al., 2007; Matano et al., 2017; Lane et al., 2015; Lane and Cohen-Gadol, 2014). Indeed, perforating arteries from the anterior communicating artery, internal carotid artery, and basilar artery, were better visualized by FNa videoangiography rather than ICG (p < 0.0001) (Matano et al., 2017). Despite its benefits, FNa videoangiography shows reduced contrast compared to ICG and persistent vessel wall staining at high doses. Its extended half-life limits its repeated use (Suzuki et al., 2007; Kakucs et al., 2017; Matano et al., 2017; Lane et al., 2015), and its inability to detect residual aneurysms and parent artery stenosis is reported in about 10% of cases (Kucukyuruk et al., 2021; Tang et al., 2002), necessitating postoperative angiography for confirmation (Derdeyn et al., 1997) or a combined use with ICG videoangiography.

### 3.2 Oncology neurosurgery

**Fig. 1:**
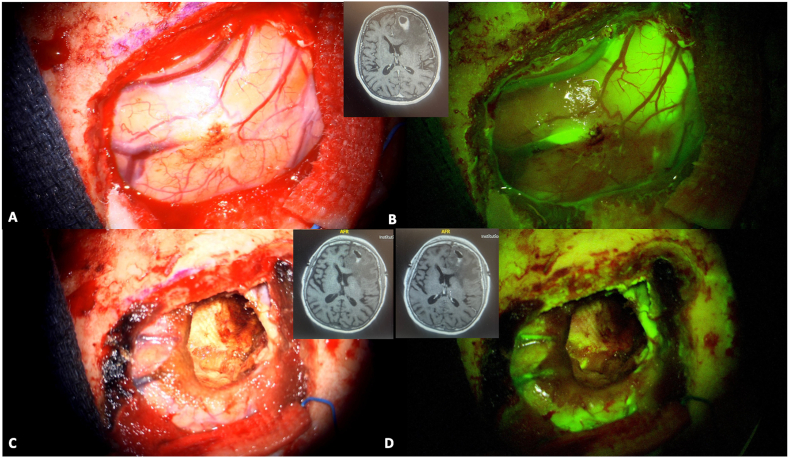
A 74 years old patient was diagnosed for a cerebral metastasis on the left frontal lobe. Intraoperative anatomical views are provided in picture A and C. A bolus of intravenous fluorescein sodium was administered at anesthesia induction (3 mg/kg) and a YELLOW 560 nm filter was used to visualize the tumor (Picture B and D). The tumor was well-defined and fluorescein sodium helped in achieving a complete resection (panel D). A preoperative is also provided (small upper panel) and compared to a postoperative MRI with and without gadolinium administration (lower right and left panel respectively). (For interpretation of the references to color in this figure legend, the reader is referred to the Web version of this article.)

Historically, FNa was the first fluorophore to be used to mark cerebral tumors. An increased GTR rate was described in FNa-guided tumor resection compared to conventional white light surgery (73.4–85.7% vs. 30.1–62.5%, respectively) (Shinoda et al., 2003; Koc et al., 2008; Chen et al., 2012; Catapano et al., 2017; Katsevman et al., 2019; Hong et al., 2019; Smith et al., 2021) and recent studies indicated that FNa may be a plausible alternative to 5-ALA for high grade gliomas (HGG) surgery (Bomers et al., 2020; Acerbi et al., 2018), as the two fluorophores resulted in comparable GTR rates (62% vs. 64%, p = 0.76, respectively) (Hansen et al., 2019). However, future studies are necessary to validate the potential pro and cons of the two dyes for HGG. Beside HGG, FNa can also be applied to the resection of other brain tumors, including medulloblastoma (Chen et al., 2022), lymphomas (Franzini et al., 2023), low-grade gliomas (Hohne et al., 2020; Minkin et al., 2016; Akcakaya et al., 2017) and brain metastases (Ahrens et al., 2022) (Fig. 1), where it has been shown to increase the rate of GTR when compared to white light surgery (Hohne et al., 2020; Okuda et al., 2010; Schebesch et al., 2015; Xiao et al., 2018; Kofoed et al., 2022; Save et al., 2019). Indeed, FNa can accumulate in all tumors with contrast enhancement on CT or MR (Schipmann et al., 2019), although its accumulation was also observed in some tumors with no contrast-enhancement (Schebesch et al., 2018), underlying that its mechanism of action may be more complex than what is currently understood. Specifically, in non-enhancing, low-grade gliomas, FNa may label focal regions of vascular dysregulation that have been correlated with high-grade features. Furthermore, FNa was useful in detecting pathological tissue with stereotactic brain biopsy, improving its diagnostic accuracy and expediting the procedure (Narducci et al., 2023; Singh et al., 2023). Also in the pediatric population, FNa seems to be a feasible and valid adjunct to recognize tumor margins with an easy-to-use and safe profile and its utility was shown with gangliogliomas and pilocytic astrocytomas (de Laurentis et al., 2023a, 2023b). If a specific YELLOW 560 nm filter integrated in the operative microscope is used, a dose between 2 and 5 mg/kg can be administered, while where no filter is used, a higher dose between 10 and 20 mg/kg is administered. In oncology procedures, the most common timing of administration is about 30–45 prior to skin incision and 1 h prior to dural opening (Minkin et al., 2016; de Laurentis et al., 2023b; Manoharan and Parkinson, 2020).

A major criticism in the use of FNa is that it is less tumor-specific than 5-ALA because it is extracellular, time limited, and fluorescence may also accumulate in perilesional edema and in areas of surgical injury (Stummer, 2015).

### 3.3 Endoscopic endonasal procedures

Intratechal FNa has been largely used to identify the site of spontaneous CSF fistula in the anterior skull base during endoscopic procedures (Seth et al., 2010; Felisati et al., 2008; Keerl et al., 2004) and it was also adopted to evaluate the rate of intraoperative CSF leak after endoscopic endonasal surgeries to tailor the most appropriate closure technique (Jakimovski et al., 2014; Placantonakis et al., 2007). It can be administered at anesthesia induction, through a lumbar tap or a lumbar drain. Commonly, about 10 ml of CSF are withdrawn and then mixed with 25 mg of FNa (e.g. 0.25 ml of 10% fluorescein solution). This solution can then be reinfused slowly (Jakimovski et al., 2014). Fluorescein identification is generally possible with a white light and the use of filters does not seem to add much to the visualization of CSF leak.

Scarce reports investigated the usefulness of intravenous FNa in assisting resection of PitNETs or other sellar tumors (da Silva et al., 2010; Romano-Feinholz et al., 2019; Bongetta et al., 2021). Functioning PitNET seem to show the highest level of fluorescence in a microscopic-assisted surgical series, where a bolus of 8 mg/kg was administered approximately 10 min before the durotomy (Romano-Feinholz et al., 2019). Bongetta et al. described a full-endoscopic case of Cushing disease, where a weight-based dose of 3 mg/kg of FNa was administered iv soon after the dural opening (Bongetta et al., 2021): the normal pituitary tissue was fluorescent while PitNET did not show any fluorescence, similar to the contrast enhancement at cerebral MRI after gadolinium administration (Bongetta et al., 2021). The different timing of administration could explain this variability in fluorescence. However, evidences on the usefulness of iv FNa in PitNET resection remains very limited (Vergeer et al., 2022; Bongetta et al., 2021).

• **5-Aminolevulinic acid (5-ALA)**

5-Aminolevulinic acid (5-ALA) is the biochemical precursor of protoporphyrin IX (PpIX), which represents the penultimate compound in heme synthesis, before the enzymatic addition of Fe2+ by ferrochelatase (Ahrens et al., 2022). Notably, PpIX tends to accumulate in tumoral cells, due to the diminished presence of ferrochelatase (Lakomkin and Hadjipanayis, 2018) and 5-ALA applications are thus limited to neuro-oncology. PpIX absorbs light from the blue spectrum (375–440 nm) and emits a red–pink light (640–710 nm) upon relaxation. This is only discernible under microscopic observation when employing a specialized filter, known as the BLUE 400 filter (Picart et al., 2019; Tonn and Stummer, 2008). Although there is a slight overlap between excitation and emission spectra, the normal brain appears blue in contrast to the intense red fluorescence typically emitted by tumoral tissue (Picart et al., 2019). However, intraoperative use of 5-ALA requires darkening of the operating room, complicating simultaneous visualization of normal neural and vascular structures that should be preserved. Consequently, surgeons must frequently alternate between light conditions to ensure both safety and benefit from 5-ALA fluorescence. Despite the advantages in surgical visualization of tumoral tissue, the use of 5-ALA presents certain drawbacks limiting its broader applications. Primarily, its relatively high cost; secondly, the necessity of oral administration 3–4 h prior to surgery poses logistical challenges; thirdly, the compound has phototoxic side effects persisting for 24 h, necessitating precautions to prevent patient exposure to sunlight or intense white light to avoid sunlight-induced erythema (Senders et al., 2017). Other adverse events are rare and minor according to literature analysis and include nausea, vomiting and gastroesophageal reflux (Picart et al., 2019; Cordova et al., 2016). Anemia, thrombocytopenia and leukocytosis, along with a biological rise in transaminases and cholestatic enzymes was described the first day after 5-ALA administration (Stummer et al., 2006). Candidates should be carefully selected as patients with porphyria, kidney and hepatic diseases, along with pregnant and breast-feeding patients, are contraindicated to receive 5-ALA (Guyotat et al., 2016).

### 3.4 Oncology neurosurgery

At present, 5-ALA stands as the sole sanctioned medication for fluorescence guided surgery for HGG and it represents the gold standard. It is considered as a tumor-specific fluorophore and is generally administered orally at a dosage of 20 mg/kg, 3 h prior to surgery (Picart et al., 2019; Stummer et al., 2006, 2017; Gandhi et al., 2019; Haj-Hosseini et al., 2015). Nevertheless, recent investigations revealed that peak fluorescence is observed approximately 7–8 h post-administration, extending even further in marginal tumor tissue, up to 8–9 h (Kaneko et al., 2019). After oral ingestion, the bioavailability is highly efficient in HGG cells, secondary to the breakdown of the BBB, which is typically impermeable to 5-ALA, and to the heightened expression of membrane ABC-transporters in tumor cells, both facilitating the entry of 5-ALA (Zhao et al., 2013). It should be kept in mind that, as fluorescence is dependent from the amount of disruption of the BBB, some antiepileptics and steroids can interfere with 5-ALA visualization (Mazurek et al., 2022). Stummer et al. emphasized the significance of administering dexamethasone before surgery (3 doses of 4 mg over 2 days) to enhance the uptake of 5-ALA, reduce the efflux of PpIX and decrease peritumoral fluorescence by strengthening the BBB (Stummer et al., 2006, 2017). Additionally, pre-operative treatment with steroids may help in identifying patients who may be at risk of experiencing neurological deficits following 5-ALA-guided surgery as the persistence of neurological deficits may suggest that they are caused by tumor infiltration rather than edema (Stummer et al., 2006; Guyotat et al., 2016) and the resection should be tailored accordingly.

5-ALA is only reliably absorbed in HGGs and most of the studies performed in literature focus on GTR and subsequent improvement of OS and PFS in HGG patients operated with 5-ALA administration when compared to conventional white light surgery. A recent meta-analysis described GTR rates of 79.1% with 5-ALA use compared to 52.8% without 5-ALA (Gandhi et al., 2019); the OS was increased by three months (p < 0.001) and PFS by one month (p < 0.001) (Gandhi et al., 2019). Other papers confirmed the improvement of the prognosis of patients with HGG (Stummer et al., 2006; Diez Valle et al., 2014; Coburger et al., 2015; Della Puppa et al., 2017). However, these results were not confirmed by the recent RESECT study, where GTR was significantly increased in the 5-ALA group but no impact was reported on OS and PFS (Picart et al., 2024) and its real impact on patients’ prognosis remains debated.

**Fig. 2:**
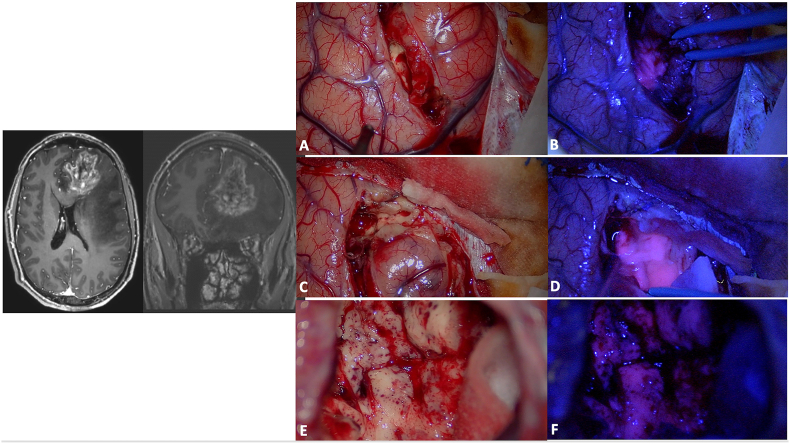
Surgical resection of a high-grade glioma in the left frontal lobe (preoperative MRI on the left; A, C and E: intraoperative anatomical views). The high-grade portion presented a superficial part at the level of the superior frontal gyrus and then extended in the depth till the frontal horn of the lateral ventricle. During the initial phase of surgical resection, the use of a dedicated BLUE 400 filter may help in identifying the tumor that shows a strong pink color, while the surrounding brain remains blue (B and D). Towards the end of the resection, the identification of normal tissue under white light may be difficult (E) and 5-ALA fluorescence may guide the surgeons in identifying residual tumor (F: residual tumor is still present at the bottom of the surgical cavity and is easily recognized by its pink color). A careful hemostasis should be performed to allow a proper visualization of the fluorophore as the blood could mask fluorescent tissue. Furthermore, it should be kept in mind that the peripheral portion of the tumor may be less fluorescent and that low grade portions may be false negative. Furthermore, 5-ALA resection should be accompanied by a careful anatomical study of preoperative images, and coupled with the use of intraoperative imaging and/or electrophysiological evaluations in eloquent areas. (For interpretation of the references to color in this figure legend, the reader is referred to the Web version of this article.)

The level of emitted fluorescence may vary in gliomas according to the histopathological characteristics of the tumor and it seems proportional to the proliferative index (Labuschagne, 2020). It was discovered that the expression of Heme Oxygenase 1 protein acts as a suppressor of 5-ALA-induced fluorescence in HGG cells and recent findings indicate that the presence of EGFR variant III affects the function of this enzyme, thus impacting cellular fluorescence (Fontana et al., 2017). Moreover, tumors with methylated MGMT are more likely to exhibit fluorescence in (Coburger et al., 2017). During tumor resection, surgeons can distinguish three different areas: a necrotic portion, showing no fluorescence, a solid tumor region, displaying intense red fluorescence (both easily discernible under white light), and infiltration zones, characterized by a pink fluorescence visible solely under blue light (Fig. 2). The red fluorescence corresponds to areas highlighted on contrast-enhanced MRI scans, while the pink fluorescence corresponds to regions of non-contrast-enhancement at MRI (Picart et al., 2019). On the same line, in gliomas with no contrast enhancement, 5-ALA use could facilitate intra-operative identification of anaplastic foci (Widhalm et al., 2010; Ewelt et al., 2011). Some technical challenges may be associated with 5-ALA guided surgery as the procedure can be time-consuming and the operative field should be kept as dark as possible, trying to reduce exposure to white light (Navarro-Bonnet et al., 2019), even if normal brain photosensitivity is debated (Stummer et al., 1998, 2006, 2017; Guyotat et al., 2016). Moreover, appropriate hemostasis should be performed to avoid that blood covering masks fluorescent areas (Guyotat et al., 2016). Other false negatives can be found at the periphery of the tumor (Hadjipanayis et al., 2015; Lau et al., 2016; Panciani et al., 2012; Stummer et al., 2014) and in necrotic areas (Hadjipanayis et al., 2015; Kiesel et al., 2018), where tumor cell density is decreased.

5-ALA usefulness in recurrent tumors was scarcely investigated (Nabavi et al., 2009; Quick-Weller et al., 2016; Kamp et al., 2015; Chohan and Berger, 2019; Hickmann et al., 2015). Globally the benefit of 5-ALA-FGS seems to be similar in recurrent and primary HGG (Picart et al., 2019) and its use could be an independent predictor of increased OS also in recurrent cases (Hickmann et al., 2015). However, the sensitivity of 5-ALA fluorescence detection may be decreased, as the differentiation between tumor and normal brain tissue may be more difficult, secondary to treatment effects (Schupper et al., 2021). On the other side, false positive cases may be found with reactive astrocytes in recurrent and previously irradiated tumors (Hadjipanayis et al., 2015; Panciani et al., 2012; Stummer et al., 2014; Kamp et al., 2015), choroid plexus or ependymal wall (Moon et al., 2016).

During the last decade, with the spreading of awake craniotomy techniques, the debate opened for 5-ALA application for tumors located in eloquent or near-eloquent areas. A concern exists that the use of 5-ALA may lead to more radical resections resulting in a higher rate of neurological complications (Stummer et al., 2006). It is clear that 5-ALA does not provide any functional information and its use should be associated with other tools to obtain the best onco-functional balance and reduce the risk of neurological deficits (Guyotat et al., 2016). However, literature data show that the increase in neurological morbidity seems to be transient, and by 3 months the difference seems to be no longer significant, with a general health status improved in the 5-ALA group (Kim et al., 2014; Picart et al., 2017). Besides the risk of side effects and technical requirements, the high cost of a dose of 5-ALA should be considered: studies assessing the cost-effectiveness of 5-ALA-FGS, concluded that it was cost-effective for the resection of HGG (Picart et al., 2019, 2024; Mansouri et al., 2016; Senft et al., 2012; Jenkinson et al., 2018).

Concerning other histological subtypes, 5-ALA showed promising results in infiltrative meningiomas, that seems to be highly fluorescent (Valdes et al., 2014; Coluccia et al., 2010; Cornelius et al., 2014). On the other side, 5-ALA usefulness in low grade gliomas or metastases remains unclear (Ahrens et al., 2022; Bianconi et al., 2023) and this limits its applications in general neuro-oncology surgery (Schupper et al., 2021).

### 3.5 Endoscopic endonasal procedures

The application of 5-ALA in pituitary surgery remains debated. Some authors evaluated the use of intraoperative 5-ALA fluorescence to identify PitNET using a protocol similar to what is performed for gliomas (20 mg/kg administered 3 h before surgery), with contrasting results (Eljamel et al., 2009; Marbacher et al., 2014). Less that 10% of PitNET showed some faint fluorescence, while the others were negative (Marbacher et al., 2014; Micko et al., 2020). A multicentric study also reported a limited interest in the use of 5-ALA for other anterior skull base pathologies that could be addressed through endoscopic approaches, namely meningiomas, craniopharyngiomas, Rathke cleft cysts, esthesioneuroblastomas, and sinonasal squamous cell carcinomas (Micko et al., 2020). In conclusion, literature data are scarce and there is no solid evidence to recommend the use of 5-ALA for endoscopic endonasal procedures as most of the pathologies treated through these approaches do not show fluorescence.

• **Indocyanine green (ICG)**

The first applications of indocyanine green (ICG) were in the assessment of hepatic function and in the performance of ophthalmic angiography (Senders et al., 2017). In neurosurgery, microscope-integrated ICG angiography was firstly adopted in 2003 (Raabe et al., 2003, 2005a). Since then, its applications expanded from vascular neurosurgery to open oncological procedures and endoscopic procedures (Kim et al., 2011; Schubert et al., 2010; Ferroli et al., 2011a). Unlike 5-ALA and FNa, the peak excitation and emission of ICG is in the near-infrared (NIR) region of the light spectrum (805 nm and 835 nm respectively), invisible to the naked eye (Hilgard et al., 1993). Indeed, ICG requires specialized equipment for detection (NIR camera or IR800 filter) (Zhao et al., 2019), with a complete dark background (Raabe et al., 2005b). The key advantages of this fluorophore lie in the binding of ICG to the vascular system via plasma proteins, thus allowing its specific use for intraoperative visualization of real-time cerebral perfusion. Furthermore, its low toxicity and rapid excretion, predominantly into the bile (Catapano et al., 2018), make ICG a safe choice with the possibility of multiple administrations during the same procedure. Indeed, ICG half-life is about 3–4 min and thus its administration can be easily repeated after 5–10 min. An *intraoperative bolus* can be used as single dose and intraoperative dosages are timing-sensitive for optimal results (Vergeer et al., 2022). A *second-window technique* can also be adopted and seems to be particularly informative during tumoral surgery or endoscopic endonasal procedures. In this setting, ICG is administered as an infusion 24 h before surgery (Cho et al., 2018), assuming that tumor vasculature presents an enhanced permeability and thus ICG would be retained in tumor cells. However, this is not a tumor-specific phenomenon, as it is also observed in necrotic and inflammatory tissue.

ICG side effects are rare: minor adverse reactions (nausea, skin rash) were observed in 0.2% of cases, while hypotension, arrhythmia and anaphylactic shock were observed in 0.05% of cases (Cochran et al., 2001).

### 3.6 Vascular neurosurgery

**Fig. 3:**
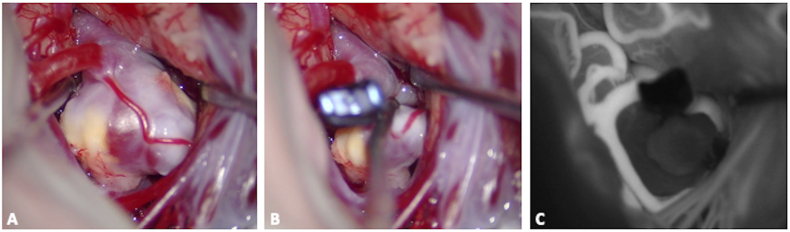
Surgical clipping of a middle cerebral artery aneurysm on the right side. After the dissection of the sylvian fissure, the aneurysm was identified, along with M1 and the superior and inferior divisions (M2), Panel A. Once the definitive clip was positioned at the aneurysm neck (Panel B), an intraoperative bolus of ICG helped in establishing the complete exclusion of the aneurysm (black) along with the adequate filling of M1 and of the two M2 (white; Panel C). This procedure, coupled with intraoperative doppler evaluations, can limit the incidence of parent arteries' and bifurcation's stenosis and of residual filling of the aneurysm.

**Fig. 4:**
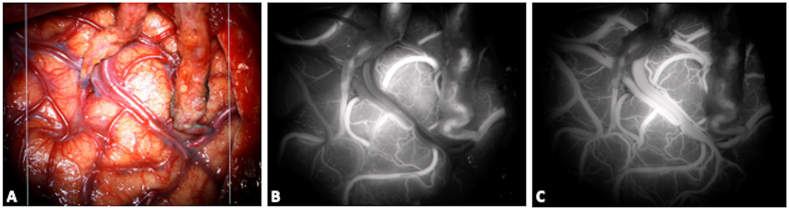
Double barrel bypass: the frontal and the parietal branch of the STA were anastomosed with two M4 arteries belonging to the superior and inferior MCA division respectively (A, anatomical view). An intraoperative bolus of ICG was administered showing an optimal permeability of the two bypasses (B: early phase of the injection; C: late phase, with an associated venous enhancement).

**Fig. 5:**
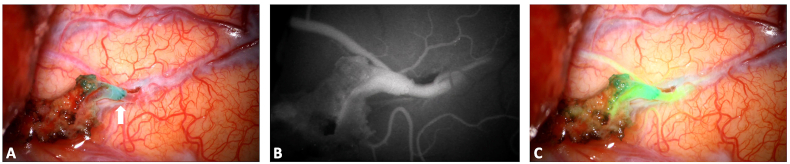
Extracranial-intracranial anastomosis on the left side for a patient with a Moya disease: the permeability of the STA-M4 bypass is checked with an intraoperative administration of indocyanin green bolus. A: Anatomical visualization of the different structures. The parietal branch of the left STA is used as donor artery, while the receiving artery is a temporal M4 branch. The white arrow indicated the anastomosis. B: Microscope-integrated ICG-videoangiography shows the classical black and white image and allows evaluation of bypass permeability. C: Augmented-reality systems integrated into the microscope may allow a real-time evaluation of blood flow while superimposing colored ICG image with the anatomical 3D view. (For interpretation of the references to color in this figure legend, the reader is referred to the Web version of this article.)

**Fig. 6:**
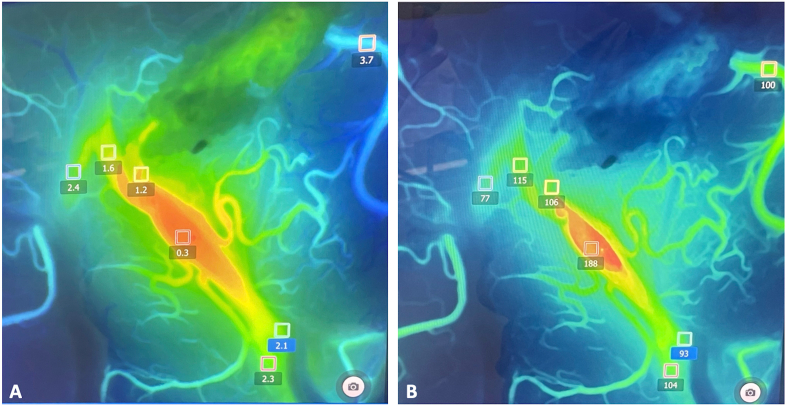
Through the use of a flow 800 software, the delay map (A, in seconds) and speed map (B, in mm/second) of ICG videoangiography can also be recorded and analyzed. In this case, they were used to evaluate bypass functionality along with flow direction and cortical perfusion. They can also be applied to the surgical resection of arterio-venous malformations or during the treatment of arterio-venous fistulae (not shown).

Despite being adopted later than FNa in vascular neurosurgery (Raabe et al., 2003), ICG is now more commonly used in fluorescent videoangiography, with the purpose of confirming the patency of parent and perforating arteries, and the adequacy of aneurysm exclusion after clipping. During the last decades, its applications in vascular neurosurgery have significantly expanded, including the evaluation of bypass permeability, AVM and DAVF flow and exclusion, and evaluation of cortical perfusion (Scerrati et al., 2014; Zaidi et al., 2014; Ng et al., 2013). Intraoperative boluses of ICG are commonly used in vascular neurosurgery and a 0.2–0.5-mg/kg intravenous bolus of ICG can be injected to obtain vessel visualization in less than a minute. ICG offers better contrast between injected vessels and surrounding structures than FNa but it requires specialized filters for its detection (Zhao et al., 2019), with a complete dark background (Fig. 3, Fig. 4). Black-and-white computer-generated videos are created (Fig. 3, Fig. 4): the relationship between the vessels and surrounding anatomy cannot be easily demonstrated (Raabe et al., 2005b) and the performance of real-time dissection during ICG videoangiography is precluded (Kucukyuruk et al., 2021). Additionally, the lower resolution of ICG videoangiography videos, especially at higher magnifications and in deep operative fields, may impede the visualization of small structures like perforating arteries (Matano et al., 2017), a phenomenon known as chromatic aberration, attributable to the limitations of the camera (Lane et al., 2015). Despite these drawbacks, ICG videoangiography remains a valuable asset in vascular neurosurgery, and technological advances can nowadays offer crucial insights into intraoperative blood flow dynamics with image overlays (Fig. 5, Fig. 6).

**Fig. 7:**
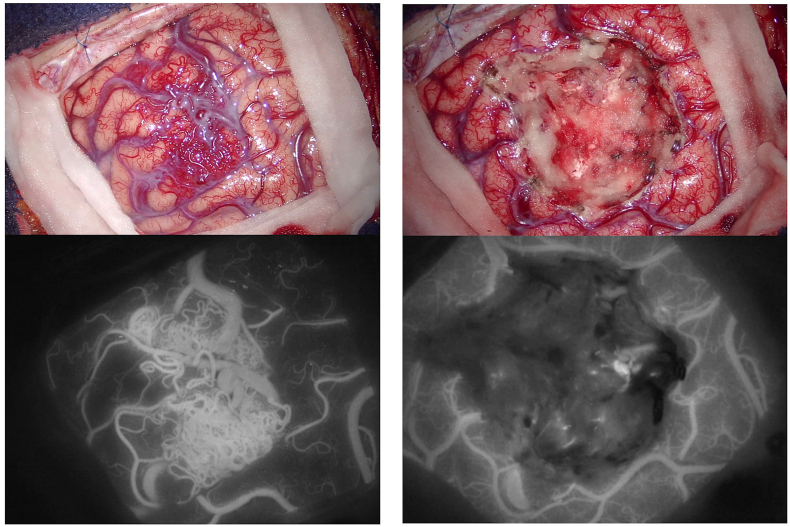
Intraoperative view of a superficial parietal arteriovenous malformation (superior parietal lobule). Intravenous ICG videoangiography may help in identifying arterial feeders, nidus and venous drainage (left panels), while helping in differentiating them from normal veins, that should be preserved. At the end of the resection a repeated ICG injection may help in establishing the completeness of the resection of the nidus (right panels).

**Fig. 8:**
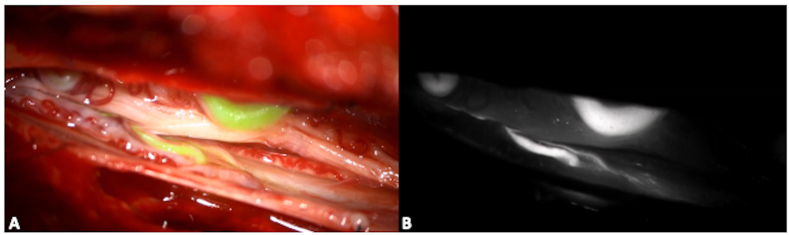
Intraoperative image of a spinal dural arterio-venous fistula. Intraoperative ICG images help in identifying the arterial feeder(s) and the draining vein(s), to guide the surgical exclusion. While black and white images show high contrast but a limited anatomical resolution (B), the superposition of color images allow real time manipulation and dissection (A). (For interpretation of the references to color in this figure legend, the reader is referred to the Web version of this article.)

In aneurysm surgery, the adequate position of the clip can be assessed in real-time: ICG can prevent stenosis of parent vessels and ischemic complications, as well as the need for revision surgeries for the presence of a residual dome (Fig. 3) (Kucukyuruk et al., 2021; Raabe et al., 2003; Dashti et al., 2009, 2011; de Oliveira et al., 2007). In a large series using ICG-videoangiography, surgical clips were repositioned during the same procedure in 9% of cases because of occlusions of parent vessels or perforating arteries, while in 4.5% of cases a supplementary clip was added to completely occlude the neck (Roessler et al., 2014). During cerebral revascularization procedures, bypass permeability and functionality may be directly evaluated (Fig. 4, Fig. 5) and recent advancements in microscope technology have introduced augmented reality systems that overlay live ICG images onto the binocular view, enabling surgeons to evaluate blood flow with a three-dimensional view (Martirosyan et al., 2015) (Fig. 5). During AVM surgery, ICG can be useful to differentiate between arterial feeders and arterialized veins (Zaidi et al., 2014; Ng et al., 2013), to evaluate the architecture of the malformation and the completeness of resection of the nidus (Fig. 7). Intraarterial injections were also successfully used to overcome the long washout time and allow repeated injections in a short time lapse during cerebral AVM resection (Shimada et al., 2020). ICG was also described for the treatment of cranial and spinal DAVF (Fig. 8) or pial AV fistulae, in the identification of hemangioblastoma feeders and during surgery for cavernous malformations, in order to delineate and preserve the adjacent venous structures (Fontes et al., 2013; Endo et al., 2013). Further application of ICG reside in endoscopic-assisted vascular procedures, as the endoscope allows a longer fluorescence visualization (10 times longer), different viewing angles, and closer view of vessels and vascular malformations (Mielke et al., 2014).

### 3.7 Oncology neurosurgery

ICG can have multiple applications in tumoral procedures: it can be used (1) to visualize and protect normal vessels during tumor resection (Ferroli et al., 2011a, 2011b; Della Puppa et al., 2014); (2) to identify pathological tumor vessels or (3) to mark the tumor itself (Catapano et al., 2018; Martirosyan et al., 2016; Germans et al., 2010). For normal vessels evaluation, the same protocol described in the vascular section can be adopted. Instead, the application of ICG to tumoral evaluation has some peculiarities that depends on the type of tumor considered. Indeed, in tumoral surgery, ICG is generally administered according to the second window technique, which allows passive accumulation of ICG in tumoral tissue (Cho et al., 2018). ICG is administered at a dose of 5 mg/kg 24 h before surgery as it progressively accumulates in HGG, pineal tumors, hemangioblastomas, meningiomas and intraventricular tumors, thanks to the enhanced permeability retention effect (Lee et al., 2016). According to a preliminary study, the same protocol was efficiently applied to cranial nerve schwannomas, to improve tumor resection and spare the nerve (Muhammad et al., 2024). An intermediary situation is represented by hemangioblastomas, where ICG is in general used to evaluate feeding vessels before tumor resection and can be administered according to the vascular protocol (Tamura et al., 2012; Hao et al., 2013).

However, the single injection technique was also described in tumoral cases: Catapano et al. used ICG in their series of brain tumors, administering a single 25 mg bolus of ICG after dural opening and before tumor removal to assess tumor margins and to visualize tumoral and peritumor vascularization, using an endoscope with a dedicated filter (Catapano et al., 2018).

### 3.8 Endoscopic endonasal procedures

ICG can have versatile applications in endoscopic endonasal procedures: its first application was performed by Litvack et al., in 2012 through the use of a standard endoscope with a near-infrared light source and an excitation wavelength filter (Litvack et al., 2012).

Intravenous boluses of 12.5–25 mg can be injected twice or thrice during surgery. ICG can show vascular structures up to 3–10 mm below the tissues’ surface, such as the septal artery under the mucosa and the paraclival ICAs under the bone (Alander et al., 2012; Yokoyama et al., 2016). The localization of parasellar ICAs and superior and inferior intercavernous sinuses can be analyzed before dura opening with a single injection during a standard endoscopic approach (Hide et al., 2015). In extended approaches instead, two doses could be administered: the first one during the nasal step to identify the septal artery and tailor the nasoseptal flap. The second administration could be performed just before the durotomy to visualize vascular structures epidurally (Catapano et al., 2018). Indeed, fluorescence can confirm the vascular anatomical information acquired through the intraoperative neuronavigation and ICG fluorescence is supposed to be superior than doppler in the detection of ICA position (Simal Julian et al., 2016). Furthermore, before durotomy ICG may help in visualizing dura invasion because of the higher vascularity of the dura in this area (Litvack et al., 2012).

**Fig. 9:**
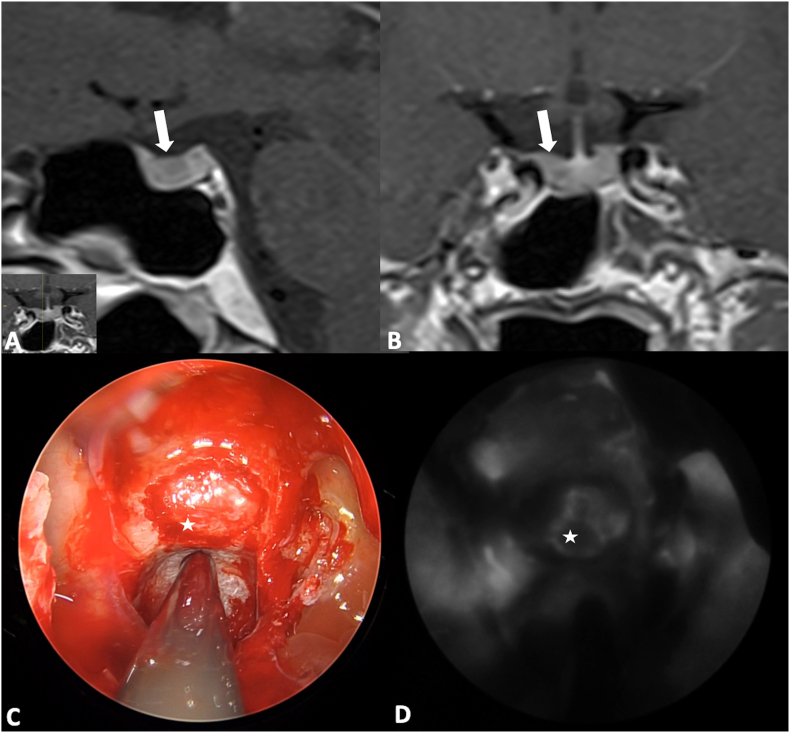
Preoperative T1-weighted MRI of a patient with a Cushing disease, after gadolinium administration (A and B). The ACTH-secreting micro pituitary neuro-endocrine tumor (PitNET) was visualized on the right portion of the pituitary gland, as assessed in the sagittal (A) and coronal plane (B) of the MRI (white arrow). The tumor showed less contrast enhancement than the normal pituitary gland and it appears as hypointense on this sequence. An intraoperative picture is provided, showing the dura mater of the sella turcica after bone removal (C). The aspiration in the clival recess. A bolus of ICG is then administered and the contrast enhancement is visualized through the dura as it strongly accumulates in the normal pituitary gland and less in the tumor (D). This confirms that the PitNET, marked with a white star, is localized on the right side of the sella and a selective approach can be performed, guided by fluorescence.

During PitNET resection a delayed-window ICG technique was described to distinguish the normal pituitary gland from the tumor between 15 and 90 min (peak) (Muto et al., 2023). After ICG administration, the normal pituitary gland remains fluorescent, allowing its differentiation and preservation during tumor resection (Catapano et al., 2018). This is independent from the hormonal production of PitNET (Fig. 9).

Furthermore, endoscopic fluorescence visualization is significantly prolonged (10 times longer, till 40 min of visualization) when compared to the microscopic traditional technique (Catapano et al., 2018; Mielke et al., 2014).

According to the same principles, for craniopharyngioma surgery, ICG can help assessing the position of pituitary stalk at the beginning of surgery and then its integrity at the end of surgery and thus predict the occurrence of endocrinological deficits after surgery (Makino et al., 2023).

Critical points in the use of ICG are the need to obtain a very clear surgical field with no bleeding, to avoid false positive images and the necessity of performing a continuous switch between normal light and fluorescent images during the endoscopic procedure.

• **Specific fluorophores for endoscopic endonasal procedures: OTL38**

OTL38 (On Target Laboratories, West Lafayette, Indiana) is a folate analog conjugated with a cyanine dye, conceived to target folate receptor alpha (FRα) (Zhang et al., 2019), that is selectively overexpressed in non-functioning PitNET (Lakomkin and Hadjipanayis, 2018). This fluorophore has an excitation wavelength of 785 nm, and an emission wavelength spectrum of 800–835 nm, being classified as NIR-fluorophore (Vergeer et al., 2022). Its application should be formally restricted to non-functioning PitNETs (Cho et al., 2018, 2019), even if Lee et al. also observed some degree of fluorescence in tumors not over-expressing FRα. This was probably secondary to an enhanced permeability of the tumoral vasculature, thus allowing a passive accumulation of the marker when compared to the normal tissue that could clear OTL38 faster (Lee et al., 2018). Further studies are necessary to validate the application of this specific fluorophore.

## 4 Discussion

Since 1948, when FNa was for the first time used in the field of oncological neurosurgery (Moore et al., 1948), fluorophore applications were further explored and they are nowadays routinely used. In vascular procedures, they are essential tools in assessing in real-time the surgical results. In oncology and endoscopic procedures, their role is likely to continue to expand in guiding surgeons toward maximal resections (Ahrens et al., 2022), along with the development of new detection devices. However, to perform safe oncological procedures, they should be integrated in a larger surgical armamentarium, including intraoperative imaging techniques such as navigation, ultrasound and/or MRI, along with electrophysiological monitoring, to evaluate the anatomical boundaries of the tumors and possible functional boundaries of the resection.

Future perspectives may emerge through two distinct ways: focusing on innovating technologies to improve the detection of existing fluorophores, and developing new fluorophores, more specific and potentially less toxic. Technical developments include high-resolution imaging systems, sensitive detection methods to obtain a higher signal to noise ratio, and more sophisticated data analysis algorithms to extract maximum information from fluorescence signals. For instance, since fluorescence intensity with 5-ALA seems to correlate with the proliferation index and considered the fact that 40% of non-fluorescent tumor biopsies still have PpIX concentrations >0.1 ng/ml, techniques of quantitative ex-vivo spectroscopy were developed (Stummer et al., 2014; Valdes et al., 2011). In the near future, this could help in optimizing resection of HGG (Picart et al., 2019; Montcel et al., 2013). Endoscopic techniques may of course help in visualizing fluorescence around blind spots that would be missed by conventional microscopic techniques and integrated filters are becoming common in recent endoscopes, to switch from white light to a selected filter to identify fluorescent areas. Furthermore, dual-axis confocal microscopes were developed (Sanai et al., 2011) with the possibility of using hand-held probes (endomicroscopy) to improve the analysis of the resection cavity in the detection of tumor infiltration at glioma margins (Xu et al., 2024). Fluorophores such as 5-ALA (Sanai et al., 2011; Pavlov et al., 2016; Wei et al., 2017) and FNa (Martirosyan et al., 2016; Belykh et al., 2018) may increase the sensitivity and specificity of the technique, help in analyzing resection cavities and increase the accuracy of biopsies (Pavlov et al., 2016). Recent data also support the use of photodynamic therapy by light-activation of 5-ALA, with encouraging results (Vermandel et al., 2021).

On the other side, advancing new fluorophores involves research and development efforts aimed at creating novel molecules with enhanced properties, such as improved brightness, photostability, and spectral characteristics. BLZ-100 (tozuleristide, Blaze Bioscience Inc, Seattle, WA), also commonly known as “tumor paint,” is a conjugate of NIR with the tumor-specific peptide chlorotoxin (Patil et al., 2019), that is extracted from scorpion venom and binds to the cell surface of gliomas through the binding to matrix metalloproteinase-2 and annexin A2. A recent phase I study showed safety for its application in patients with primary and recurrent glioblastoma (Patil et al., 2019). Furthermore, BLZ-100 could also mark cavernous malformations according to preliminary data (Kobets et al., 2021). Similarly, alkylphosphocholine analogs are small synthetic phospholipid ether molecules, with a prolonged intracellular retention and preclinical studies showed how they can label glioblastoma cells with high selectivity (Swanson et al., 2015). EGF-receptors has been shown to be expressed on their wild type or mutated form on the cell surface of glioblastoma (Senders et al., 2017). Anti-EGFR antibodies, coupled with fluorescent dyes could be used to specifically target tumoral tissue (Davis et al., 2010). On the same line, Cetuximab, an EGFR inhibitor, when conjugated with fluorescent dye (IRDye 800), was used to identify glioblastoma tissue, with an optimal signal to background ratio and adequate tumor visualization according to preliminary data (Miller et al., 2018). Recently, protease-activated probes that use NIR fluorophores showed a high efficiency in marking tumoral tissue, with a higher contrast between tumor and nontumoral brain tissue than what is obtained with 5-ALA, thanks to the activation of the probe by glioma cells and tumor-associated macrophages. Thus, they have the potential to become the targeted agent for FGS for HGG (Konecna et al., 2024).

Concerning endoscopic techniques instead, the question of the usefulness of fluorophores as part of the surgical routine for Pit-NET removal remains open. Selective labelling of PitNET tissue with fluorescent dyes is a field of active research and it could be a future solution to detect tumors, above all in cases of functional PitNET with negative MRI (Honegger and Nasi-Kordhishti, 2022).

## 5 Conclusions

A comprehensive summary of the different applications and use of the most common fluorophores is provided. Their integration in the daily neurosurgical practice can improve the prognosis and management of patients with tumors and vascular malformations. Promising researches and developing technologies will further expand their applications in the near future.

## Disclosure

All the authors approved the final version of the manuscript.

## Declaration of competing interest

None.

## ABBREVIATIONS (alphabetical order):

5-ALA: 5-Aminolevulinic acid
AVM: arterio-venous malformations
BBB: blood-brain barrier
CNS: central nervous system
CSF: cerebrospinal fluid
DAVF: dural arterio-venous fistulae
FNa: Fluorescein sodium
FGS: fluorescence-guided surgery
HGG: high grade gliomas
ICG: Indocyanine green
NIR: near-infrared
PitNET: pituitary neuroendocrine tumors
UV: Ultraviolet
